# Correction: A 3-dimensional {4^2^·8^4^} lvt net built from a ditopic bis(3,2′:6′,3′′-terpyridine) tecton bearing long alkyl tails

**DOI:** 10.1039/d2ce90094g

**Published:** 2022-07-07

**Authors:** Y. Maximilian Klein, Edwin C. Constable, Catherine E. Housecroft, Alessandro Prescimone

**Affiliations:** Department of Chemistry, University of Basel Mattenstrasse 24a BPR 1096 CH4058 Basel Switzerland catherine.housecroft@unibas.ch

## Abstract

Correction for ‘A 3-dimensional {4^2^·8^4^} lvt net built from a ditopic bis(3,2′:6′,3′′-terpyridine) tecton bearing long alkyl tails’ by Y. Maximilian Klein *et al.*, *CrystEngComm*, 2015, **17**, 2070–2073, https://doi.org/10.1039/C4CE02347A.

The authors regret an error in the assignment of the net descriptor. In the original paper, the Schläfli symbol was given as {4^2^·8^4^} leading to the assignment of an **lvt** net. The correct Schläfli symbol is {4^2^·8^4^}·{4^2^·8^4^} corresponding to a **pts** net.

Throughout the paper, including the title, **lvt** should appear as **pts**, and the assignment of {4^2^·8^4^} is corrected to {4^2^·8^4^}·{4^2^·8^4^}.

The authors apologise for this error and for any inconvenience caused to readers.

The Royal Society of Chemistry apologises for these errors and any consequent inconvenience to authors and readers.

## Supplementary Material

